# Collagen and Calcium Binding EGF Domains 1 (CCBE1) in cancer – a new role past lymphatics?

**DOI:** 10.18632/oncoscience.376

**Published:** 2017-11-09

**Authors:** Aruz Mesci, Stanley K. Liu

**Affiliations:** Sunnybrook Research Institute, Toronto, Canada; Departments of Medical Biophysics and Radiation Oncology, University of Toronto, Toronto, Canada

**Keywords:** miR-330-3p, CCBE1, breast cancer

Genes that influence cancer behavior continue to be the focus of a plethora of research studies, with the hopes of either identifying biomarkers that help predict/ prognosticate disease course, or discovering druggable targets for future therapies. Many of the gene products implicated in cancer also regulate normal developmental processes, hinting at the intimacy of the two processes. Collagen and Calcium Binding EGF Domains 1 (*CCBE1*) encodes a soluble protein with EGF-like domain and is located in human chromosomal region 18q21.32. Despite its original description as a gene frequently lost in breast cancer specimens [1], the earliest studies on the function of CCBE1 showed a role for it in lymphangiogenesis in Zebrafish [2], and in Hennekam syndrome [3]. In the last two years, more reports on the role of CCBE1 in cancer have started to emerge [4-6]. In a recent publication, we showed that CCBE1 is targeted by microRNA 330-3p (miR-330-3p), resulting in a more aggressive phenotype in breast cancer [4].

We initially observed that miR-330-3p, a tumour- promoting miR previously studied in a number of other cancers [cited in 4], was not yet investigated within the context of breast cancer. We noted that miR-330- 3p expression was elevated in a cohort of human breast cancer patients, and that its expression correlated with poorer distant relapse-free survival (DRFS). Through *in vitro* invasion assays and an *ex ovo* model of metastasis using breast carcinoma cell lines, we showed that increased expression of miR-330-3p resulted in a more aggressive phenotype. We also identified CCBE1 to be a direct target of miR-330-3p using luciferase assays, and that CCBE1 loss via siRNA-mediated knockdown recapitulated this phenotype. In agreement with our cell-based assays, we noted that CCBE1 expression was decreased in certain patient cohorts, and that low levels of CCBE1 were associated with reduced DRFS and overall survival (OS).

Conspicuously, our results were in close agreement with the initial study that examined the role of CCBE1 in cancer [5]. The authors indicated that CCBE1 was often downregulated in ovarian and breast carcinomas via hypermethylation, and that CCBE1 loss resulted in increased migration in transwell assays. CCBE1 expression was lower in ovarian carcinomas (over normal stroma), as well as in higher grade tumours. The authors showed a non-significant trend towards lower CCBE1 expression in higher FIGO stage cancers, and speculated that its loss may occur early in carcinogenesis. Our study supported this possibility in that ductal carcinoma *in- situ* (DCIS) samples had lower expression of CCBE1 compared to normal breast tissue. However, findings of decreased DRFS with lower CCBE1 expression in both studies argue that the extent of CCBE1 loss remains consequential beyond initiation of carcinogenesis.

Not all studies support a tumour-suppressor role for CCBE1, however. A separate study investigating the clinicopathological correlation of CCBE1 expression in gastrointestinal stromal tumours (GISTs) found that CCBE1 was elevated in higher risk GISTs, and that increased expression of CCBE1 predicted lower DRFS and OS [6]. Mechanistically, CCBE1 appeared to promote angiogenesis and increased resistance to imatinib *in vitro*. While at odds with our study and that of Barton, et al., the biology inherent to GISTs compared to breast or ovarian cancers may underlie these differences. Alternatively, the tumour-suppressing or tumour-promoting function of CCBE1 may be context-specific, even within cancers of the same tissue origin.

While different perspectives on the biological roles of CCBE1 are emerging, the mechanism of CCBE1 action in cancer cells, as well as within the tumour microenvironment (e.g., stroma and vasculature) have yet to be thoroughly explored. To address this, studies are investigating signaling pathways engaged by CCBE1, and the biological function of its various domains [7-8]. Only with further research into the molecular and cellular biology of CCBE1, can we expect to gain a greater understanding of its role in cancer.
